# ABCG2 impairs the activity of the aurora kinase inhibitor tozasertib but not of alisertib

**DOI:** 10.1186/s13104-015-1405-4

**Published:** 2015-09-28

**Authors:** Martin Michaelis, Florian Selt, Florian Rothweiler, Michael Wiese, Jindrich Cinatl

**Affiliations:** Institut für Medizinische Virologie, Klinikum der Goethe-Universität, Paul Ehrlich-Str. 40, 60596 Frankfurt Am Main, Germany; Centre for Molecular Processing and School of Biosciences, University of Kent, Canterbury, CT2 7NJ UK; Pharmaceutical Institute, University of Bonn, An der Immenburg 4, 53121 Bonn, Germany; Deutsches Krebsforschungszentrum (DKFZ), Klinische Kooperationseinheit Pädiatrische Onkologie (G340) and Pädiatrie III, Zentrum für Kinder- und Jugendmedizin, Im Neuenheimer Feld 280, 69120 Heidelberg, Germany

**Keywords:** ABCG2, Drug resistance, Aurora kinase inhibitor, Tozasertib, VX680, MK-0457, Alisertib, MLN8237

## Abstract

**Background:**

Recently, we have shown that the ATP-binding cassette (ABC) transporter ABCB1 interferes with the anti-cancer activity of the pan-aurora kinase inhibitor tozasertib (VX680, MK-0457) but not of the aurora kinase A and B inhibitor alisertib (MLN8237). Preliminary data had suggested tozasertib also to be a substrate of the ABC transporter ABCG2, another ABC transporter potentially involved in cancer cell drug resistance. Here, we studied the effect of ABCG2 on the activity of tozasertib and alisertib.

**Results:**

The tozasertib concentration that reduces cell viability by 50 % (IC_50_) was dramatically increased in ABCG2-transduced UKF-NB-3^ABCG2^ cells (48.8-fold) compared to UKF-NB-3 cells and vector-transduced control cells. The ABCG2 inhibitor WK-X-34 reduced tozasertib IC_50_ to the level of non-ABCG2-expressing UKF-NB-3 cells. Furthermore, ABCG2 depletion from UKF-NB-3^ABCG2^ cells using another lentiviral vector expressing an shRNA against the bicistronic mRNA of ABCG2 and eGFP largely re-sensitised these cells to tozasertib. In contrast, alisertib activity was not affected by ABCG2 expression.

**Conclusions:**

Tozasertib but not alisertib activity is affected by ABCG2 expression. This should be considered within the design and analysis of experiments and clinical trials investigating these compounds.

**Electronic supplementary material:**

The online version of this article (doi:10.1186/s13104-015-1405-4) contains supplementary material, which is available to authorized users.

## Background

The aurora kinases A, B, and C are involved in spindle apparatus organisation during cell division [1, 2]. Inhibitors of aurora kinases represent a novel class of anti-cancer drugs currently under pre-clinical and clinical investigation [1–5]. Aurora kinases have been suggested to be potential drug targets in neuroblastoma [6–15], the most frequent extracranial solid childhood tumour. About half of neuroblastoma patients suffer from high-risk disease associated with overall survival rates below 50 % despite intensive therapy [16, 17].

Recently, we showed that aurora kinases may represent targets in therapy-refractory neuroblastoma. In particular, p53 wild-type neuroblastoma cells were sensitive to aurora kinase inhibitors [15]. Notably, only a small fraction of neuroblastomas harbours p53-mutant cells [18, 19]. In addition, we confirmed previous assumptions that ABCB1 expression confers resistance to the pan-aurora kinase inhibitor tozasertib (VX680, MK-0457) [15, 20, 21]. In contrast, the activity of the aurora kinase A and B inhibitor alisertib (MLN8237) was not affected by the presence of ABCB1 (also known as P-glycoprotein or MDR1) [15]. Tozasertib was suggested to also interfere with ABCG2 (also known as BCRP) [20], another ATP-binding cassette (ABC) transporter known to be involved in cancer cell drug resistance [22], but conclusive experimental evidence has been missing. Moreover, there is no information on a possible interaction of alisertib with ABCG2 available in the public domain. Thus, we here investigated the effects of ABCG2 expression on the anti-cancer effects of tozasertib and alisertib.

## Methods

### Drugs

Tozasertib and alisertib were purchased from Selleck Chemicals (Houston, TX, USA), mitoxantrone from Gry-Pharma GmbH (Kirchzarten, Germany). WK-X-34 was synthesised as described before [23].

### Cells

The MYCN-amplified, ABCB1-negative neuroblastoma cell line UKF-NB-3 was derived from a bone marrow metastasis of a stage IV neuroblastoma patient [24] and propagated in IMDM supplemented with 10 % FBS, 100 IU/ml penicillin and 100 mg/ml streptomycin at 37 °C. Cells were routinely tested for mycoplasma contamination and authenticated by short tandem repeat profiling. Cells showing high expression of ABCG2 were established as described previously [25, 26] using the lentiviral gene ontology (LeGO) vector technology [27, 28] (http://www.LentiGO-Vectors.de).

### Viability assay

Cell viability was tested by the 3-(4,5-dimethylthiazol-2-yl)-2,5-diphenyltetrazolium bromide (MTT) dye reduction assay after 120 h incubation modified as described previously [29].

### ABCG2 depletion in UKF-NB-3^ABCG2^ cells

The LeGO-iG2 vector that we used for the expression of ABCG2 (LeGO-iG2-ABCG2) is a bicistronic vector with an internal ribosome entry site (IRES) that links the expression of the fluorescent marker gene to the expression of another gene of interest (here ABCG2) [25–29]. Previously, it was shown that the expression of genes from this bicistronic vector can be depleted by the use of a second vector encoding an shRNA against eGFP [27]. Here, we cloned the eGFP-shRNA (GCACGACTTCTTCAAGTCC [27]) into the LeGO-X vector that uses dsRedExpress (orange emission, 584 nm) as marker [27] (http://www.LentiGo-Vectors.de) resulting in the vector LeGO-X-GFP2.

### Flow cytometry

An antibody directed against ABCG2 (Kamiya Biomedical Company, Seattle, WA, USA), followed by secondary antibody labelled with Phycoerythrin (R&D, Wiesbaden, Germany) was used to detect protein expression by flow cytometry (FACSCanto, BD Biosciences, Heidelberg, Germany).

### Statistics

Results are expressed as mean ± SD of at least three experiments. Comparisons between two groups were performed using Student’s *t* test. Three and more groups were compared by ANOVA followed by the Student–Newman–Keuls test. P values lower than 0.05 were considered to be significant.

## Results

### Effects of tozasertib and alisertib on the viability of ABCG2-expressing cells

**Fig. 1:**
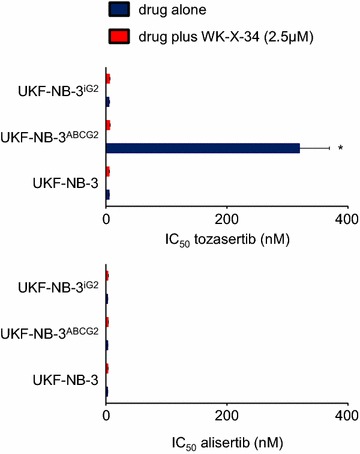
Effects of tozasertib and alisertib on the viability of non-ABCG2-expressing UKF-NB-3 cells, UKF-NB-3 cells transduced with a lentiviral vector encoding for ABCG2 (UKF-NB-3^ABCG2^), or UKF-NB-3 cells transduced with a control vector (UKF-NB-3^iG2^) in the absence or presence of the ABCG2 inhibitor WK-X-34 (2.5 µM) as determined by MTT assay after 120 h of incubation. WK-X-34 (2.5 µM) alone did not affect cell viability (Additional file 1: Table S1). *P < 0.05 relative to IC_50_ UKF-NB-3 in the absence of WK-X-34

The concentration that reduces cell viability by 50 % (IC_50_) was dramatically increased in ABCG2-transduced UKF-NB-3^ABCG2^ cells for tozasertib (48.8-fold) and mitoxantrone (a cytotoxic ABCG2 substrate that was used as control, 296.5-fold) (Fig. 1; Additional file 1: Table S1). In the presence of the ABCG2 inhibitor WK-X-34, the tozasertib and mitoxantrone IC_50_ values were reduced to the level of non-ABCG2-expressing UKF-NB-3 cells (Fig. 1; Additional file 1: Table S1). In contrast, alisertib activity was not affected by ABCG2 expression (Fig. 1; Additional file 1: Table S1).

### Effects of ABCG2 depletion on tozasertib efficacy

**Fig. 2:**
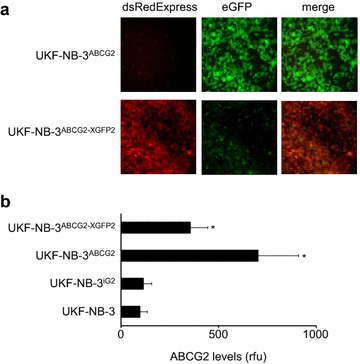
ABCG2 depletion using a second lentiviral vector (LeGO-X-GFP2) with dsRedExpress as marker encoding an shRNA targeting the bicistronic ABCG2-IRES-eGFP mRNA of the first vector (LeGO-iG2-ABCG2) thereby depleting eGFP and ABCG2 expression. **a** Fluorescence pictures indicating dsRedExpress (encoded as marker by LeGO-X-GFP2) and eGFP fluorescence in UKF-NB-3 cells transduced with LeGO-iG2-ABCG2 (UKF-NB-3^ABCG2^) or LeGO-iG2-ABCG2 and LeGO-X-GFP2 (UKF-NB-3^ABCG2-XGFP2^). **b** ABCG2 levels in UKF-NB-3 cells, UKF-NB-3 cells transduced with the empty LeGO-iG2 vector (UKF-NB-3^iG2^), UKF-NB-3^ABCG2^ cells, or UKF-NB-3^ABCG2-XGFP2^ cells as determined by flow cytometry and expressed as relative fluorescence units (rfu). *P < 0.05 relative to UKF-NB-3

**Fig. 3:**
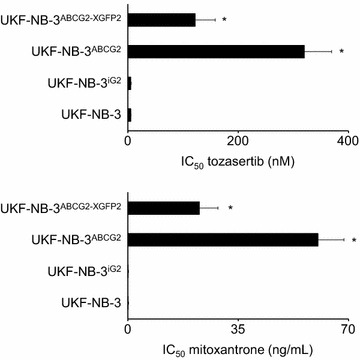
Effects of ABCG2 depletion on UKF-NB-3^ABCG2^ cell sensitivity to tozasertib and the cytotoxic ABCG2 substrate mitoxantrone. Concentrations that reduce cell viability by 50 % after 120 h incubation (IC_50_) were determined by MTT assay in UKF-NB-3 cells, UKF-NB-3 cells transduced with a control vector (UKF-NB-3^iG2^), UKF-NB-3 cells transduced with the lentiviral vector LeGO-iG2-ABCG2 encoding for ABCG2 (UKF-NB-3^ABCG2^), and UKF-NB-3^ABCG2^ cells in which ABCG2 was depleted using a lentiviral vector encoding an shRNA directed against the mRNA of eGFP and ABCG2 (LeGO-X-GFP2) of the LeGO-iG2-ABCG2 vector (UKF-NB-3^ABCG2-XGFP2^). *P < 0.05 relative to UKF-NB-3

In order to deplete ABCG2 from UKF-NB-3^ABCG2^ cells, we additionally transduced these cells with the LeGO-X-GFP2 vector encoding an shRNA directed against eGFP. Fluorescence microscopy indicated effective reduction of eGFP protein levels in UKF-NB-3^ABCG2-XGFP2^ cells (Fig. 2a). Moreover, flow cytometric analysis demonstrated decreased ABCG2 levels in UKF-NB-3^ABCG2-XGFP2^ cells (Fig. 2b). In accordance with the results from the use of the ABCG2 inhibitor WK-X-34, UKF-NB-3^ABCG2-XGFP2^ cells were re-sensitised to tozasertib and the cytotoxic ABCG2 substrate mitoxantrone (Fig. 3).

## Discussion

Knowledge about the interaction of drug candidates with ABC transporters is important for their investigation and (pre-)clinical development because ABC transporters are expressed at organ and tissue barriers determining drug body distribution [30]. Moreover, ABCG2 expression may be involved in cancer cell drug resistance [22].

Previously, we had shown that the pan aurora kinase inhibitor tozasertib that is a frequently used tool compound [with 128 articles in the Pubmed (http://www.ncbi.nlm.nih.gov/pubmed) as of 19th August 2015] but not the aurora kinase A and B inhibitor alisertib that substantially differs in structure from tozasertib and is under investigation in multiple clinical trials ([3–5], 50 clinical studies of alisertib are registered at http://www.clinicaltrials.gov as of 19th August 2015) interferes with ABCB1-mediated drug transport [15]. Here, we provide evidence that the efficacy of tozasertib is also affected by ABCG2 expression. ABCG2 expression reduced cancer cell sensitivity to tozasertib and the cytotoxic ABCG2 substrate mitoxantrone. Interference with ABCG2 using WK-X-34, an ABCG2 inhibitor, or RNAi-mediated ABCG2 depletion resulted in re-sensitisation of ABCG2-expressing cells to tozasertib (and mitoxantrone). This is in concordance with previous findings suggesting an interaction of tozasertib with ABCG2 [20] although conclusive experimental evidence had been missing. Cancer cell lines adapted to the aurora kinase inhibitor AZD1152 had been shown to express high levels of ABCG2 and to be cross-resistant to tozasertib [20]. However, studies confirming that there is a functional relationship between high ABCG2 expression and decreased tozasertib sensitivity had not been performed. Moreover, this is the first study that investigated a potential effect of ABCG2 on the activity of alisertib and provides evidence that ABCG2 expression does not impair the efficacy of alisertib.

In conclusion, the differential effects of ABCG2 on tozasertib and alisertib activity should be carefully considered within the design and analysis of experiments and clinical trials investigating these compounds.

## Availability of supporting data

The data sets supporting the results of this article are included within the article and its Additional file 1: Table S1.

## Additional file

**Additional file 1:** **Table S1.** Effects of tozasertib, alisertib, and the cytotoxic ABCG2 substrate mitoxantrone on the viability of non- ABCG2-expressing UKF-NB-3 cells, UKF-NB-3 cells transduced with a lentiviral vector encoding for ABCG2 (UKF-NB-3ABCG2), or UKF-NB-3 cells transduced with a control vector (UKF-NB-3piG2) in the absence or presence of the ABCG2 inhibitor WK-X-34.

## Abbreviations

MTT: 3-(4,5-dimethylthiazol-2-yl)-2,5-diphenyltetrazolium bromide
ABC transporter: ATP-binding cassette transporter
IC_50_: concentration that reduces cell viability by 50 %
FBS: foetal bovine serum
IMDM: Iscove’s modified Dulbecco’s medium
