# Supplementary material for: ABCG2 impairs the activity of the aurora kinase inhibitor tozasertib but not of alisertib
Source: BMC Res Notes. 2015 Sep 28;8:484. doi: 10.1186/s13104-015-1405-4 (PMC4587578; doi:10.1186/s13104-015-1405-4)
Supplement: Supplementary file 1 — Additional file 1: Table S1. Effects of tozasertib, alisertib, and the cytotoxic ABCG2 substrate mitoxantrone on the viability of non- ABCG2-expressing UKF-NB-3 cells, UKF-NB-3 cells transduced with a lentiviral vector encoding for ABCG2 (UKF-NB-3ABCG2), or UKF-NB-3 cells transduced with a control vector (UKF-NB-3piG2) in the absence or presence of the ABCG2 inhibitor WK-X-34. [file 13104_2015_1405_MOESM1_ESM.pdf]

Suppl. Table 1. Effects of tozasertib, alisertib, and the cytotoxic ABCG2 substrate mitoxantrone on the viability of non-ABCG2-expressing UKF-NB-3 cells, UKF-NB-3 cells transduced with a lentiviral vector encoding for ABCG2 (UKF-NB-3^{ABCG2}), or UKF-NB-3 cells transduced with a control vector (UKF-NB-3^{piG2}) in the absence or presence of the ABCG2 inhibitor WK-X-34 as determined by MTT assay after 120h of incubation.

	IC_{50}[1] tozasertib (nM)	+ WK-X-34 (2.5µM)	
		WK-X-34 alone[2]	
UKF-NB-3	5.50 ± 0.22	97 ± 2	5.52 ± 0.34 (1.0)[3]
UKF-NB-3^{ABCG2}	319.7 ± 49.6	95 ± 3	6.55 ± 0.64 (48.8)
UKF-NB-3^{piG2}	5.31 ± 0.82	96 ± 2	6.12 ± 0.69 (0.9)
	IC_{50}[1] alisertib (nM)	+ WK-X-34 (2.5µM)	
		WK-X-34 alone[2]	
UKF-NB-3	2.94 ± 0.43	97 ± 2	3.49 ± 0.29 (0.8)[3]
UKF-NB-3^{ABCG2}	3.13 ± 0.21	95 ± 3	3.94 ± 0.35 (0.8)
UKF-NB-3^{piG2}	2.86 ± 0.46	96 ± 2	3.50 ± 0.61 (0.8)
	IC_{50}[1] mitoxantrone (ng/mL)	+ WK-X-34 (2.5µM)	
		WK-X-34 alone[2]	
UKF-NB-3	0.15 ± 0.09	97 ± 2	0.18 ± 0.05 (0.8)[3]
UKF-NB-3^{ABCG2}	68.2 ± 8.2	95 ± 3	0.23 ± 0.07 (296.5)
UKF-NB-3^{piG2}	0.16 ± 0.04	96 ± 2	0.14 ± 0.04 (1.1)

[1] concentration that reduces cell viability by 50% relative to non-treated control
[2] cell viability (%) relative to non-treated control
[3] fold change IC_{50}/ IC_{50} in the presence of WK-X-34
